# Secret Remedies

**Published:** 1892-05-21

**Authors:** 


### The Doctor's Armchair.

### SECRET REMEDIES.

A distinguished ecclesiastic of the Church of Scotland told the writer the other day that during a recent visit he had paid to a university professor his host had suffered acutely from neuralgia. The visitor suggested the propriety of calling in a physician; but the learned professor avowed a great want of faith in doctors. He believed a little in hydropathy, he said, and in some other more or less sectarian methods of that kind; but in the regular practitioner he confessed he had very little faith. The visitor, though himself an ecclesiastic, had studied medicine and surgery for a couple of years; and having thus learnt to trust medicine and surgery with sincere loyalty, he was much surprised that so learned a person as a university professor should hold what he felt to be such prejudiced and irrational opinions. He found on enquiry that there was one ground, and one only, on which the professor based his judgment. The ground was that he had seldom in his life required to consult medical men, and really knew nothing at all about them.

In the matter of "secret remedies" a state of things almost identical with what we have just recorded is pretty generally prevalent. A large section of the public blame the medical profession very severely for what they consider its attitude towards outsiders and their "specifics" and "cures." If a particular remedy will really cure a particular disease, what can it matter, say these critics, whether it were introduced into practice by orthodox or heterodox means? "And so say all of us," doctors as well as adverse critics. If a remedy will really cure we do not care one straw who introduced it into practice. There is not a medical man in the world who would willingly let his patients suffer for want of a particular remedy, whether that remedy were orthodox or unorthodox.

The point, however, we wish to make at the present time is that the public is not really aware of the actual attitude of the medical profession towards secret remedies. That attitude is entirely honourable to medical men, and one which, if universally known, would meet with universal approval. Let us take an ordinary manufacturer—say, of the ink for the printing of this newspaper, or of the paper on which it is printed. That manufacturer most likely has "trade secrets." It is considered by everybody to be quite proper that he should have them, and that he should make as much money as possible out of them. If he be given to advertising, he emphasises in his advertisement the value of his trade secret; and every interested person thinks him specially lucky in having such a secret, and specially "smart" in that he utilises it so cleverly in the building up of a large fortune.

But now, gentlemen of commerce, you who are so ready to smile pityingly upon the doctor as a narrow-minded and etiquette-governed specialist, give a little heed whilst we compare your behaviour with that of the honourable medical practitioner! Is it not easy to see and understand that the doctor, as well as the manufacturer has every possible opportunity for the acquisition of "trade secrets?" In the matter of drugs alone he has the choice of more than twelve hundred different substances. What infinite possibilities of combination there are in these twelve hundred different drugs! Then again, as regards varieties of personal experience! One physician may see more before he is forty years of age than another may see in a life time. Here are unparalleled opportunities for the accumulation of still more trade secrets. Why should not a shrewd doctor, like a wide-awake manufacturer, take care of his own secrets, and grow rich by them? The medical man has surely as much right to the fruit of his own brains and labour as the manufacturer! Is it not easy to see that if Sir Andrew Clark or Mr. Jonathan Hutchinson were to announce the discovery of a special remedy for gout, or cancer, or consumption, and to advertise his discovery in every newspaper in the civilised world, a gigantic fortune would almost immediately flow in; a fortune compared with which Holloway's half-million would be a mere drop in the bucket?

Such are the obvious possibilities of wealth which lie open to the practitioner of more than average reputation. But does any physician or surgeon of eminence ever yield to these golden temptations? This is the attitude of all honourable medical men towards secret remedies; they will not grow rich by them; they will not use them; they will not countenance them. They unitedly and voluntarily contract themselves out of the use of them. Nay more, they mutually and heartily agree that, if the knowledge of any new or secret remedy comes into their possession, they will at once make it known, and that, without charge, to all their medical brethren, for the benefit of the whole world.

Now it seems to the writer that a fair appeal may here be made to the reason and justice of a generally fair-minded public. Is it an honourable thing, or is it not, for every member of the medical profession to throw open the whole of his knowledge and the whole of his experience to all the other members without fee or reward? It seems to us splendidly honourable, even religiously honourable.

But here is the almost comical irony of the situation: whilst the legitimate doctor, who has spent hundreds of pounds and years of time in acquiring his licence to practice, voluntarily cuts himself off from the profits of secret remedies, an outsider who is no doctor at all, and who has never spent either money or time in acquiring a knowledge of the medical art, may come in, and, by the diligent puffing of a pretended secret remedy, acquire a fortune. Not only so, but the honourable medical man, who has freely given away all the knowledge which he has acquired by his diligent studies, is abused and sneered at by a discriminating (?) public because he does not honour and sit at the feet of the dishonourable impostor who gets rich by arts that he himself is too generous to practise. The situation, from the point of view of reason and fairness, is supremely ridiculous. It is as if a man should give away his coat to a beggar, and all the parish should turn round and applaud the beggar for having a coat, but deride the giver because he does not walk arm-in-arm with the beggar up the High Street into the bargain. One thing at least seems obviously fair: if the duly qualified practitioner does not use secret remedies, the unqualified certainly ought not to be allowed to use them. If the great unqualified insist upon practising medicine, they should at least be compelled to practise it openly. That simple rule the public ought certainly to insist upon, if only for the preservation of its own skin.

